# *UBTF::ATXN7L3* gene fusion defines novel B cell precursor ALL subtype with CDX2 expression and need for intensified treatment

**DOI:** 10.1038/s41375-022-01557-6

**Published:** 2022-04-09

**Authors:** Lorenz Bastian, Alina M. Hartmann, Thomas Beder, Sonja Hänzelmann, Jan Kässens, Miriam Bultmann, Marc P. Hoeppner, Sören Franzenburg, Michael Wittig, Andre Franke, Inga Nagel, Malte Spielmann, Niklas Reimer, Hauke Busch, Stefan Schwartz, Björn Steffen, Andreas Viardot, Konstanze Döhner, Mustafa Kondakci, Gerald Wulf, Knut Wendelin, Andrea Renzelmann, Alexander Kiani, Heiko Trautmann, Martin Neumann, Nicola Gökbuget, Monika Brüggemann, Claudia D. Baldus

**Affiliations:** 1 Medical Department II, Hematology and Oncology, University Medical Center Schleswig-Holstein, Kiel, Germany; 2 University Cancer Center Schleswig-Holstein, University Medical Center Schleswig-Holstein, Kiel and Lübeck, Germany; 3 Clinical Research Unit “CATCH-ALL” (KFO 5010/1), funded by the Deutsche Forschungsgemeinschaft (DFG, German Research Foundation), Bonn, Germany; 4 Institute for Clinical Molecular Biology, Kiel University, Kiel, Germany; 5 Institute of Human Genetics, University Medical Center Schleswig-Holstein, Kiel & Lübeck, Germany; 6 DZHK (German Centre for Cardiovascular Research), Partner Site Hamburg/Lübeck/Kiel, 23538 Lübeck, Germany; 7 Medical Systems Biology Group and Institute for Cardiogenetics, University of Lübeck, Lübeck, Germany; 8 University Hospital Schleswig-Holstein, Campus Lübeck, Lübeck, Germany; 9 Department of Hematology, Oncology and Tumor Immunology (Campus Benjamin Franklin), Charité-Universitätsmedizin Berlin, corporate member of Freie Universität Berlin and Humboldt-Universität zu Berlin, Berlin, Germany; 10 Department of Medicine II, Hematology/Oncology, Goethe University Hospital, Frankfurt/M, Germany; 11 Department of Internal Medicine III, University Hospital Ulm, Ulm, Germany; 12 Department of Hematology, Oncology and Clinical Immunology, University Hospital Düsseldorf, Düsseldorf, Germany; 13 Department of Hematology and Oncology, University Hospital Göttingen, Göttingen, Germany; 14 Medical Department V, Hospital Nürnberg, Paracelsus Medizinische Privatuniversität, Nürnberg, Germany; 15 Medical Department Oncology and Hematology, University Medical Center Oldenburg, Oldenburg, Germany; 16 Department of Medicine IV, Hematology/Oncology, Klinikum Bayreuth, Bayreuth, Germany; 17 Comprehensive Cancer Center Erlangen-EMN (CCC ER-EMN), Erlangen, Germany

**Keywords:** Oncogenesis, Cancer genomics

## To the Editor:

Genomic aberrations—gene fusions in the majority of cases—and corresponding transcriptional regulations define an increasingly complex landscape of molecular subtypes in B cell precursor acute lymphoblastic leukemia (BCP-ALL) [1]. Up to 15% of patients cannot be allocated to established subtypes, suggesting the presence of unrecognized drivers—especially in adult patients who have been less studied so far.

**Fig. 1:**
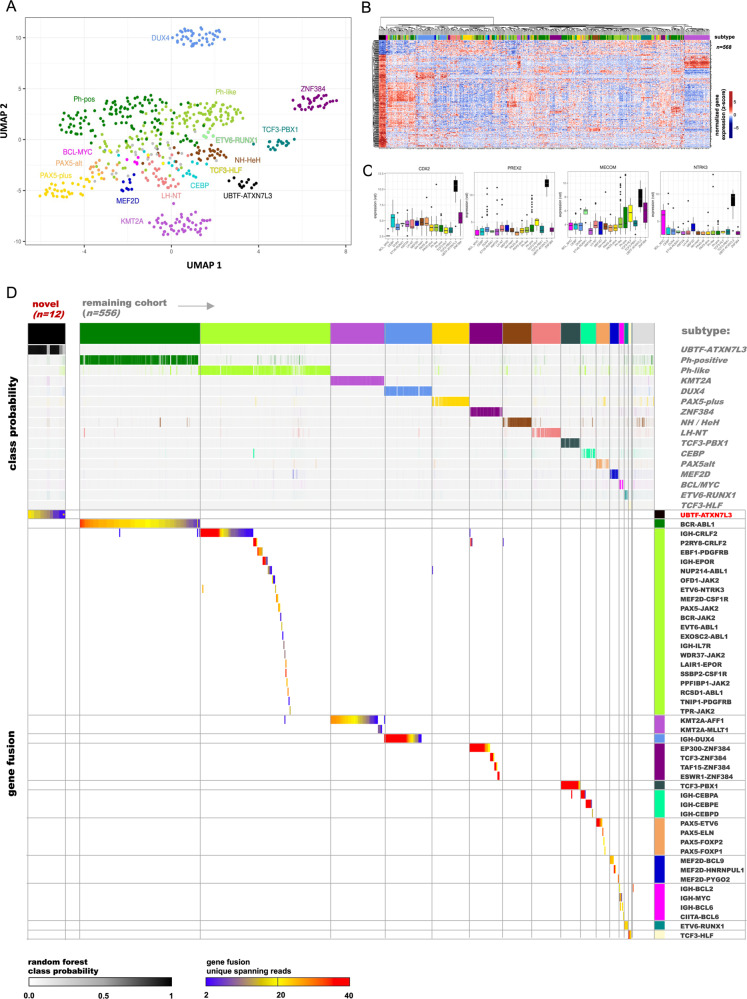
*UBTF::ATXN7L3* gene fusion defines a BCP-ALL molecular subtype. Bone marrow or peripheral blood samples of *n* = 568 adult patients at first diagnosis of BCP-ALL with at least 20% blast infiltration were analyzed by transcriptome sequencing. Gene expression profiles of previously established definitions [1] were used to train a machine learning algorithm (Extreme Gradient Boosting) with feature selection (LASSO) to allocate samples to 15 established and 2 novel BCP-ALL subtypes. **A** Uniform manifold approximation and projection (UMAP) plot depicts distribution of molecular ALL subtypes based on expression of the top informative genes for subgroup allocation. **B** Unsupervised clustering of gene expression specific for the novel UBTF::ATXN7L3 subgroup. Subtype-specific gene sets were identified by multi-comparison ANOVA using the variance stabilizing transformation for normalizing expression values. Genes had to be differentially expressed to at least 11 other subtypes with an FDR-corrected *p* value ≤ 0.01 (Supplementary Table S1,2). **C** Expression of selected UBTF::ATXN7L3-specific oncogenes is shown in comparison to other molecular subtypes with at least three samples. The complete list of Cosmic cancer gene census genes differentially expressed by multi-comparison ANOVA in UBTF::ATXN7L3 ALL is shown in Supplementary Table S2. Expression of cancer-associated genes upregulated in UBTF::ATXN7L3 in comparison to other molecular subtypes is shown in Supplementary Figure S6. **D** Subtype-specific driver fusions and the novel candidate driver gene fusion *UBTF::ATXN7L3* were called from RNA-Seq data (FusionCatcher) [15] Virtual karyotypes were obtained from whole-exome sequencing or SNP-Arrays (Illumina Infinium Global Screen array v3.0) to support classification of aneuploid subtypes. Subtype-specific hotspot driver mutations (eg. *PAX5*) were called from RNA-Seq data. Heatmap depicts probabilities for allocation of samples to the molecular subtypes (class probabilities) obtained by the gene expression-based machine learning classifier together with corresponding genomic driver events. * indicates one UBTF::ATXN7L3 sample with 20% blast content, where the driver gene fusion was not called by FusionCatcher but was confirmed by break-point specific PCR and sanger sequencing (Supplementary Fig. S7B).

**Fig. 2:**
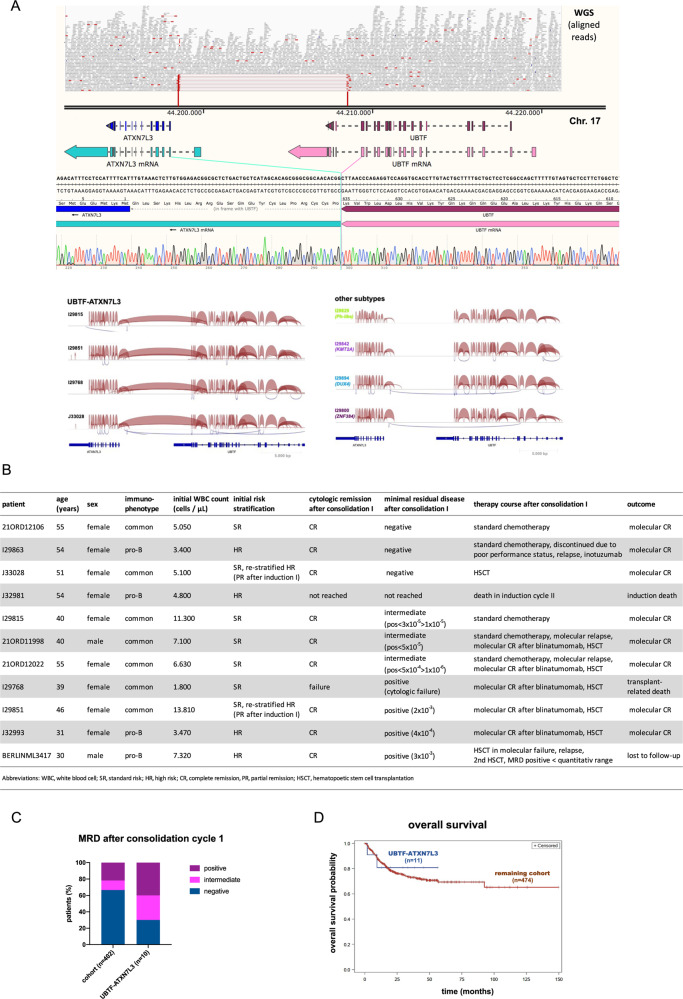
UBTF::ATXN7L3 ALL patients experience poor responses to conventional chemotherapy and successful salvage by MRD-stratified treatment intensification including immunotherapies. **A** The structure of *UBTF::ATXN7L3* fusion is shown. Above: whole-genome sequencing read alignment from one representative sample (I29815) with reads depicted as pairs and red highlighting insert lengths above the 99.5 percentile of all reads; middle: NCBI reference sequence of 17q21.31 (chr17:44,191,805-44,221,804); below: sanger sequence of a break-point specific PCR on cDNA and sashimi plots depicting junction reads from RNA-Seq data of representative UBTF::ATXN7L3 patients and representative patients from other subtypes. **B** Basic clinical characteristics, treatment courses and outcome of UBTF::ATXN7L3 patients are shown. **C** Minimal residual disease (MRD) was measured by clone-specific real-time quantitative PCR of immune gene rearrangements after consolidation cycle I. Negative: MRD negative with sensitivity of at least 10^−4^, positive: MRD above 10^−4^ also including cytologic non-response, intermediate: MRD positive < 10^−4^ or below quantifiable range. **D** Overall survival analysis of UBTF::ATXN7L3 patients compared to the remaining cohort.

We performed transcriptome sequencing (RNA-Seq) on *n* = 568 adult BCP-ALL patients prospectively treated according to pediatric-based protocols of the German Multicenter Acute Lymphoblastic Leukemia (GMALL) study group including risk stratification based on minimal residual disease (MRD) and treatment intensification for high-risk patients. To define molecular subtypes, we used our previous integrative analyses [1] to train a machine learning classifier to predict subtype allocation from gene expression profiles of subsequently sequenced samples. Feature selection (LASSO) was used to identify the most informative genes. Underlying genomic aberrations were analyzed (whole-genome sequencing (WGS), whole-exome sequencing (WES); SNP-arrays) to confirm subtype allocation in selected cases. With this approach, we were able to allocate *n* = 535/568 (94%) samples to 15 previously established [1] molecular subtypes (Fig. 1A–D), with confirmation of corresponding genomic alterations in 91% of analyzed cases (Fig. 1D). Unsupervised gene expression analysis of previously unassigned samples revealed a distinct patient subset (*n* = 12; Supplementary Fig. S1A) defined by a novel in-frame gene fusion of upstream binding transcription factor (*UBTF*) and ataxin-7-like protein 3 (*ATXN7L3*) occurring exclusively in this patient cluster (*n* = 12/12 vs. *n* = 0/556 in remaining cohort, *p* < 1E−10; Fig. 1D). Comparison of gene expression profiles revealed that *UBTF::ATXN7L3* rearranged cases in our cohort match to a recently described BCP-ALL subtype, which so far was identified by increased expression of the homeobox transcription factor CDX2 (‘CDX2 high’ ALL) [2] (Supplementary Fig. S1B). *UBTF::ATXN7L3* represents an 11.3 kbp in-frame read-through between *UBTF* exon 17/21 and a 5′ UTR splice site of *ATXN7L3*, with the same sanger sequencing confirmed break point in all samples (Fig. 2A, Supplementary Methods). WGS of 3 samples revealed a 10.08 kb genomic deletion involving *UBTF* 3′ exons (18-21) and most of the intergenic region between UBTF and ATXN7L3 as underlying mechanism (Fig. 2A, Supplementary Fig. S2). Break-point-specific PCR and Sanger sequencing confirmed presence of the deletion in *n* = 11/11 UBTF::ATXN7L3 patients with available material (Supplementary Fig. S3). The same *ATXN7L3* transcript breakpoint has previously been identified in a patient with diffuse large B cell lymphoma (*GPATCH8*::*ATXN7L3*) [3], suggesting a shared driver function in different B-lymphoid malignancies. Both fusion partners were highly expressed across the entire cohort without significant differences in the novel subtype (Supplementary Fig. S4), suggesting either gain-of-function or a dominant-negative effect of the gene fusion.

Both, UBTF and ATNX7L3 are global epigenetic regulators involved in transcriptional control. UBTF is an essential co-activator of ribosomal RNA expression. Very recently, UBTF has been characterized as novel oncogene in acute myeloid leukemia (AML), where internal tandem duplications define a distinct molecular subtype with poor outcome and highest incidence in early adolescents [4, 5]. WGS and sanger sequencing ruled out UBTF internal tandem duplications in UBTF::ATXN7L3 patients (data not shown). ATXN7L3 is a global gene expression co-activator through the SAGA complex. It is essential for activation of the SAGA histone deubiquitinase module (DUBm) through USP22, which is part of the 11-gene signature “Death-from-cancer” [6] defining poor outcomes across entities. The SAGA DUBm competes for ATXN7L3-binding with other deubiquitinases suggesting global changes in gene expression upon imbalances in ATXN7L3-substrate binding [7]. These findings align well with data on other molecular ALL subtypes driven by epigenetic perturbations [8, 9]. Analysis of subtype-specific gene expression by multi-comparison ANOVA revealed 332 genes with differential expression in UBTF::ATXN7L3 ALL when compared to each other subtype (Fig. 1B; Supplementary Tables S1 and 2). These differentially expressed genes included upregulation of 18 cancer-associated genes (COSMIC Cancer gene census, Supplementary Table S3), one of which was *CDX2*, which has been used to define ‘CDX2-high’ ALL [2] (Fig. 1C). However, few samples from other subtypes also showed increased *CDX2* expression levels, limiting its applicability to identify this subtype. UBTF and ANTX7L3 are global epigenetic regulators without described functional interactions with CDX2. *CDX2* is expressed in AML [10] and ALL [11], independently of the driver subtype. Conditional *Cdx2* overexpression in hematopoietic progenitors resulted in myelodysplasia but required acquisition of secondary aberrations for leukemic transformation [12], suggesting a cooperative function during leukemogenesis. Although UBTF::ATXN7L3-specific gene expression showed little overlap with published *CDX2* overexpression models (Supplementary Fig. S5A), we identified a functional module relating *CDX2* to *HOXA9* and *MEIS1* overexpression in UBTF::ATXN7L3 ALL, in line with similar findings in AML [13] (Supplementary Figure S5B,C). HOXA9/MEIS1 are essential co-factors for KMT2A-driven leukemogenesis [9], making it possible that a CDX2-HOXA9/MEIS1 axis exerts a similar leukemia promoting role in UBTF::ATXN7L3 ALL. Further oncogenes related to hematologic malignancies were also upregulated in UBTF::ATXN7L3 patients (Fig. 1C, Supplementary Figure S6), including NTRK3 which might represent a therapeutic target for specific inhibitors (e.g. larotrectinib, entrectinib). To evaluate additional genomic driver aberrations, we performed WES (*n* = 7) and/or SNP-array analyses (n = 6) showing a described enrichment of chromosome 1q gains [2] (*n* = 5/7) and heterogeneous single chromosome aberrations. However, no subtype-specific recurrent driver events were identified (Supplementary Fig. S7A), supporting the functional relevance of *UBTF::ATXN7L3* as recurrent hallmark of this subtype. UBTF::ATXN7L3 ALL was enriched for pro-B immunophenotypes (*n* = 5/12, 42% vs. *n* = 70/530, 13%; *p* = 0.016) and occurred predominantly in female patients (*n* = 10/12, 83% vs. 237/534, 44%; *p* = 0.008) and patients of advanced age (median: 48.5 years vs. 38 years; *p* = 0.05).

Outcome evaluable UBTF::ATXN7L3 patients (*n* = 11/12; Fig. 2B) received treatments on pediatric inspired GMALL protocols. Risk stratification identified 6 patients as high-risk due to pro-B immunophenotype (*n* = 4) or late response (*n* = 2). One patient died during induction therapy and another patient failed to achieve hematologic CR after consolidation I (overall cytologic CR rate: 82%). Only *n* = 3/10 patients cleared MRD after consolidation I (cytologic and molecular CR) compared to *n* = 271/402 (67%; *p* = 0.019; Fig. 2C) in the remaining cohort. Two out of these three good responders remained in molecular CR after conventional chemotherapy including allogenic stem cell transplantation (HSCT) due to high-risk criteria. One patient relapsed after discontinuation of standard chemotherapy due to poor performance status and achieved a second molecular CR after inotuzumab ozogamizin. Patients with intermediate MRD response (positive MRD < 10^−4^ or below quantifiable range, *n* = 3) experienced molecular relapses on standard therapy, received Blinatumomab followed by HSCT and remained in long-term remission (*n* = 2/3) or achieved sustained CR on standard therapy (*n* = 1/3). Among the remaining poor responders (*n* = 4), one cytologic non-responder achieved MRD-negativity after Blinatumomab, received HSCT and died due to transplant-related complications. Two patients received Blinatumomab, achieved a molecular CR, proceeded to HSCT, and remained in long-term remission. The fourth patient received HSCT without Blinatumomab, relapsed, and achieved intermediate MRD after 2nd HSCT. Together, we observed a median overall survival probability of UBTF::ATXN7L3 patients of 80% (±12%) compared to 73% (±2%; *p* = 0.07; Fig. 2D) in the remaining cohort, which is comparable to the ongoing GMALL08/2013 study [14]. Yasuda et al [2]. reported markedly lower survival rates (pOS: 26.7%, (4.8-56.3)) in ‘CDX2-high’ patients treated in historical cohorts without MRD-based risk stratification. Together, these data suggest that UBTF::ATXN7L3 ALL represents a less chemo-sensitive disease subtype, which can be successfully salvaged by current MRD-based concepts incorporating immunotherapies and stem cell transplantation [14].

Other subtypes with poor MRD response in our cohort included ZNF384 (48.2% MRD negative after consolidation I; *p* = 0.056), Ph-like ALL (54.0% MRD negative, *p* = 0.003) and KMT2A (55.8% MRD negative, *p* = 0.127), whereas high hyperdiploid (90.9% MRD negative, *p* = 0.01) and TCF3::PBX1 (94.1% MRD negative, *p* = 0.016) subtypes showed favorable MRD responses. This heterogeneity and recently published differences in treatment outcomes of ALL subtypes when treated with or without MRD-based risk-stratification highlight the importance of evaluating the clinical course of novel molecular subgroups in the context of current treatment strategies.

Yasuda et al [2]. described a second novel BCP-ALL subtype defined by *IDH1/2* hotspot mutations (1.9% of cohort). We screened RNA-Seq data of all remaining ‘unassigned’ samples of our cohort (*n* = 22) for the described gene expression signature or IDH1/2 mutations and identified one patient harboring *IDH2* p.R140Q, which was confirmed by PCR on gDNA level, contributing to the heterogeneous frequency distribution of molecular subtypes in different BCP-ALL cohorts.

Our data identify *UBTF::ATXN7L3* resulting from a 17q21.31 variant as novel subgroup defining candidate driver fusion for the recently described ‘CDX2-high ALL’ subtype. Poor MRD response indicates reduced chemosensitivity in these patients. Our data suggest MRD-based treatment intensification using salvage immunotherapies and allogenic stem cell transplantation as a promising strategy to rescue this high-risk phenotype.

## Supplementary information

Supplemental Material and Methods

Supplementary Figures S1–S7

Supplemental Tables S1–S4
