# Supplementary material for: UBTF::ATXN7L3 gene fusion defines novel B cell precursor ALL subtype with CDX2 expression and need for intensified treatment
Source: Leukemia. 2022 Apr 9;36(6):1676–80. doi: 10.1038/s41375-022-01557-6 (PMC9162919; doi:10.1038/s41375-022-01557-6)
Supplement: Supplementary file 1 — Supplemental Material and Methods [file 41375_2022_1557_MOESM1_ESM.pdf]

Supplementary Material and Methods

Patients and routine diagnostic procedures

At first diagnosis of B cell precursor acute lymphoblastic leukemia, bone marrow or peripheral blood samples were obtained from adult patients prospectively treated according to pediatric-based protocols of the German Multicenter Acute Lymphoblastic Leukemia (GMALL) study group. Patients gave their informed consent for scientific use of their banked samples. Studies were approved by the ethical review board of the GMALL study group and the institutional ethic review board of the University Medical Center Schleswig-Holstein, Kiel, Germany. Bone marrow mononuclear cells were separated by density gradient centrifugation and DNA and RNA was extracted using standard protocols. Bone marrow cytology was evaluated in specialized local laboratories of the contributing clinical centers. Flow cytometry based immunophenotyping was performed in a central reference laboratory of the study group. A minimum of 20% blast content in bone marrow / peripheral blood was required for samples to enter analysis.

Minimal residual disease analysis

To monitor leukemia burden during therapy course, clone-specific PCR-markers of immunoglobulin / T cell receptor gene rearrangements were established at first diagnosis and used for quantitative real time PCR at subsequent treatment time points. (Brueggemann M et al. Leukemia, 2019). MRD Negative: MRD negative with sensitivity of at least 10^{-4}, positive: MRD above 10^{-4} also including cytologic non-response, intermediate: MRD positive < 10^{-4} or below quantifiable range.

Transcriptome sequencing and subgroup allocation

For transcriptome sequencing, libraries were prepped using the TruSeq RNA Library Prep kit (Illumina ©) for stranded mRNA. Libraries were sequenced using 2x100bp paired reads on an Illumina NovaSeq sequencer at a median depth of ca. 40 million reads per sample. Samples with a RIN of <6 were excluded from sequencing and quality control on raw reads was performed using FastQC (S. Andrews, Babraham Bioinformatics). Raw reads were aligned to the human genome (GRCh38.p13, ensembl annotations version 94) using STAR aligner version 2.7.9a (Dobin et al., 2013, Bioinformatics). Resulting gene counts were normalized using variant stabilization transformation and gene expression was analyzed using the R package DESeq2 version 1.32.0. Molecular Subtypes were predicted based on

gene expression using an Extreme Gradient Boosting (XGB) machine learning algorithm using the R package caret and trained on a previously classified reference cohort (Bastian et al., 2019, Leukemia). For machine learning the data was randomly split into training (9/10) and testing (1/10). On the training data Least Absolute Shrinkage and Selection Operator (LASSO) was applied for feature selection using the R package glmnet (cv.glmnet function with alpha = 1). For XGB eta, nrounds, max_depth, min_child_weight and colsample_bytree were optimized in a tune-grid whereas gamma and subsample parameters were kept constant at 0 and 1, respectively. This resulted in 216 different parameter combinations for XGB tuning. To improve generalizability, we performed a stratified randomized 10-fold cross-validation. Thereby, 90% of the data was used for feature selection, hyperparameter tuning and training of the classifiers, and 10% for testing. Aligned reads were manually checked for known subgroup-defining single nucleotide alterations. Fusion transcripts were called from raw RNAseq reads using FusionCatcher version 1.33 and ensembl human genome annotation version 104. Resulting gene fusions were filtered using a manually curated list of fusion breakpoints recurrent within the present cohort and/or described in the literature as driver fusions for ALL. Integrative Genomics Browser (IGV) version 2.4.19 (Robinson et al., 2011, Nature Biotechnology) was used and to visualize the novel UBTF-ATXN7L3 fusion junction reads.

Whole exome sequencing

For whole exome sequencing, DNA libraries were prepped using the Illumina DNA Prep for Enrichment kit in combination with the IDT xGen Exome Research Panel (v2) oligios: Libraries were sequenced 2x150 bp on NovaSeq (Illumina©) to achieve an average coverage of 120x. Remission samples with MRD <1E-04 served as germ line controls. In one case with missing germ line control, population variants were excluded using gnomAD. Reads were processed using the SAREK pipeline for whole genome-/exome sequencing analysis of germline and somatic variants (Garcia M et al. F1000 Res, 2020) to obtain somatic SNVs / Indels. Copy number variants and virtual karyotypes were generated using CNVkit (Talevich E, PLOS computational Biology, 2016).

Whole genome sequencing

Libraries for whole genome sequencing were prepared using the Illumina DNA Prep kit (Illumina, San Diego, USA) and sequenced on the NovaSeq 6000 (Illumina, San Diego, USA) using the S4 Flowcell and 2x150 bp paired-end reads. Genomic short reads were adapter-trimmed with fastP (version 0.23.1, Shen S et al. Bioinformatics 2018) and aligned against the reference genome hg38 using BWA-MEM2 (version 2.2.1, https://ieeexplore.ieee.org/document/8820962). Alignments were sorted, mate coordinates filled in and reads deduplicated (Samtools version 1.14, Danecek P et al. Gigascience 2021). Subsequent variant calling was performed using DeepVariant (version 1.3.0, Yun T et al. Bioinformatics 2021) for SNPs and INDELs and MANTA (version 1.6.0, Chen X et al. Bioinformatics 2016) to detect larger structural variations.

SNP-Arrays

To obtain virtual karyotypes and analyze CNV in selected target genes, Infinium Global Screening Array v.3.0. (Illumina ©) were processed and read according to the manufacturer's instructions. Analyses were performed using GenomeStudio v.2.0.5 (Illumina ©) for data processing and Integrative Genomics Browser (IGV) version 2.4.19 (Robinson et al., 2011, Nature Biotechnology) for visualization.

Data availability

RNAseq and WGS raw data will be made available upon request in accordance with current national and international data safety regulations. Please contact the corresponding author. Primary sequencing data have been deposited to the European Genome Phenome Archive (EGAS00001006107) and will be made available through the corresponding data access committee.

Break-point specific PCR

To confirm the *UBTF::ATXN7L3* gene fusion, the following primer pair was used for PCR on cDNA: ATTGGGGCAAACACACTCCTT / TGGCAACAGAGCGTTATCGG. Sanger sequencing confirmed the specificity of the 756 bp PCR product.

To confirm the underlying genomic deletion, the following pimer pair was used for PCR on gDNA: CCTCTTTGATACCCATGGTGCAAT / GAGCCAGAAGGAGCACTACA. Sanger sequencing confirmed the specificity of the 949 bp PCR product.
