# Supplementary material for: UBTF::ATXN7L3 gene fusion defines novel B cell precursor ALL subtype with CDX2 expression and need for intensified treatment
Source: Leukemia. 2022 Apr 9;36(6):1676–80. doi: 10.1038/s41375-022-01557-6 (PMC9162919; doi:10.1038/s41375-022-01557-6)
Supplement: Supplementary file 2 — Supplementary Figures S1–S7 [file 41375_2022_1557_MOESM2_ESM.pdf]

Supplementary Figure S1

A

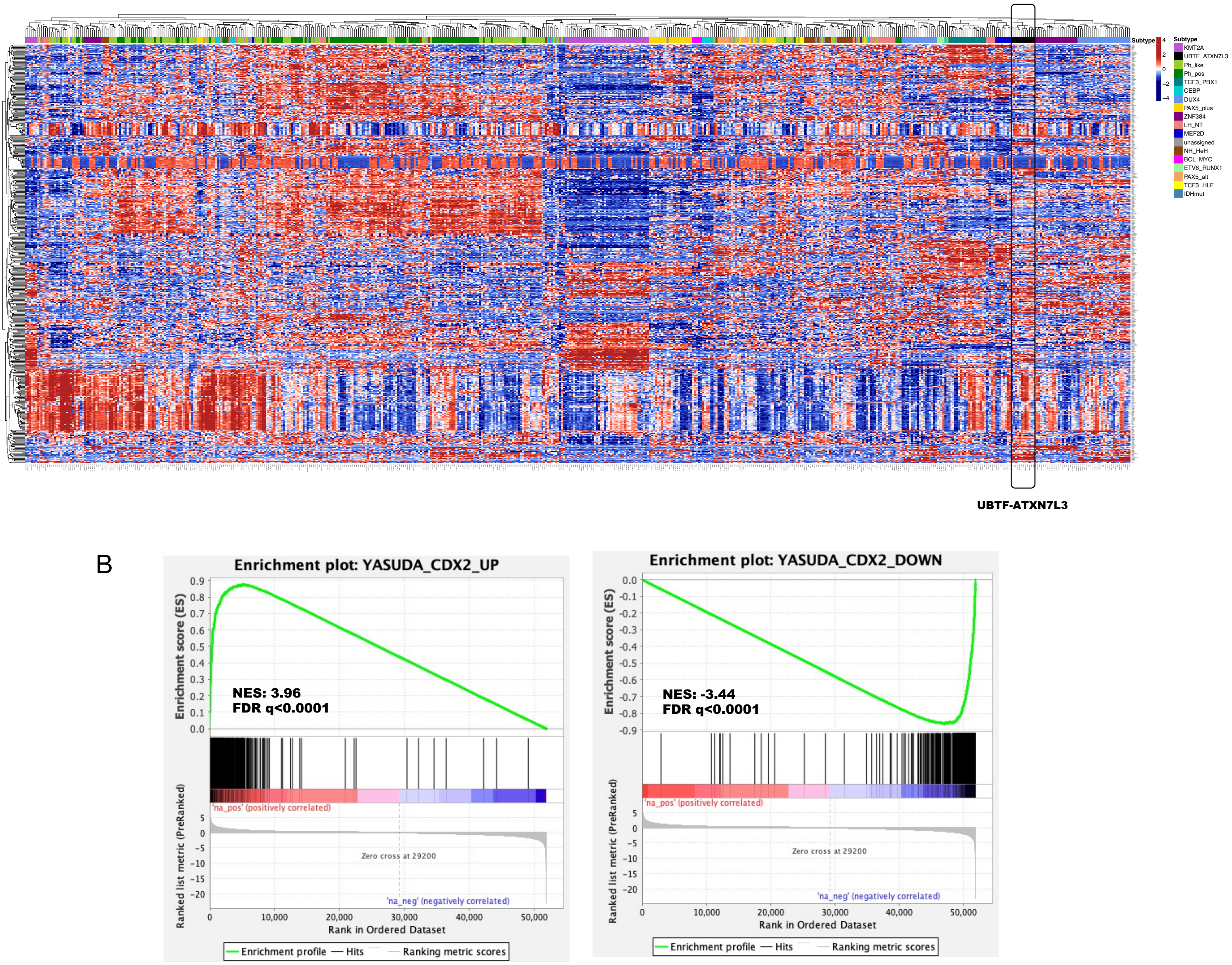

Supplementary Figure S1: (A) Heatmap depicting unsupervised clustering top variable expressed genes (n=500) in the BCP-ALL cohort shown in Main Figure 1D. UBTF-ATXN7L3 samples form a distinct cluster. **(B)** Gene set enrichment analysis using GSEA (Subramanian A et al., PNAS, 2005) of gene expression in UBTF-ATXN7L3 patients vs remaining cohort compared to gene lists of up- and downregulated genes defining ‚CDX2-high' ALL as desribed by Yasuda T et al., Blood, 2021 (NES: normalized enrichment score, FDR: false discovery rate)

A

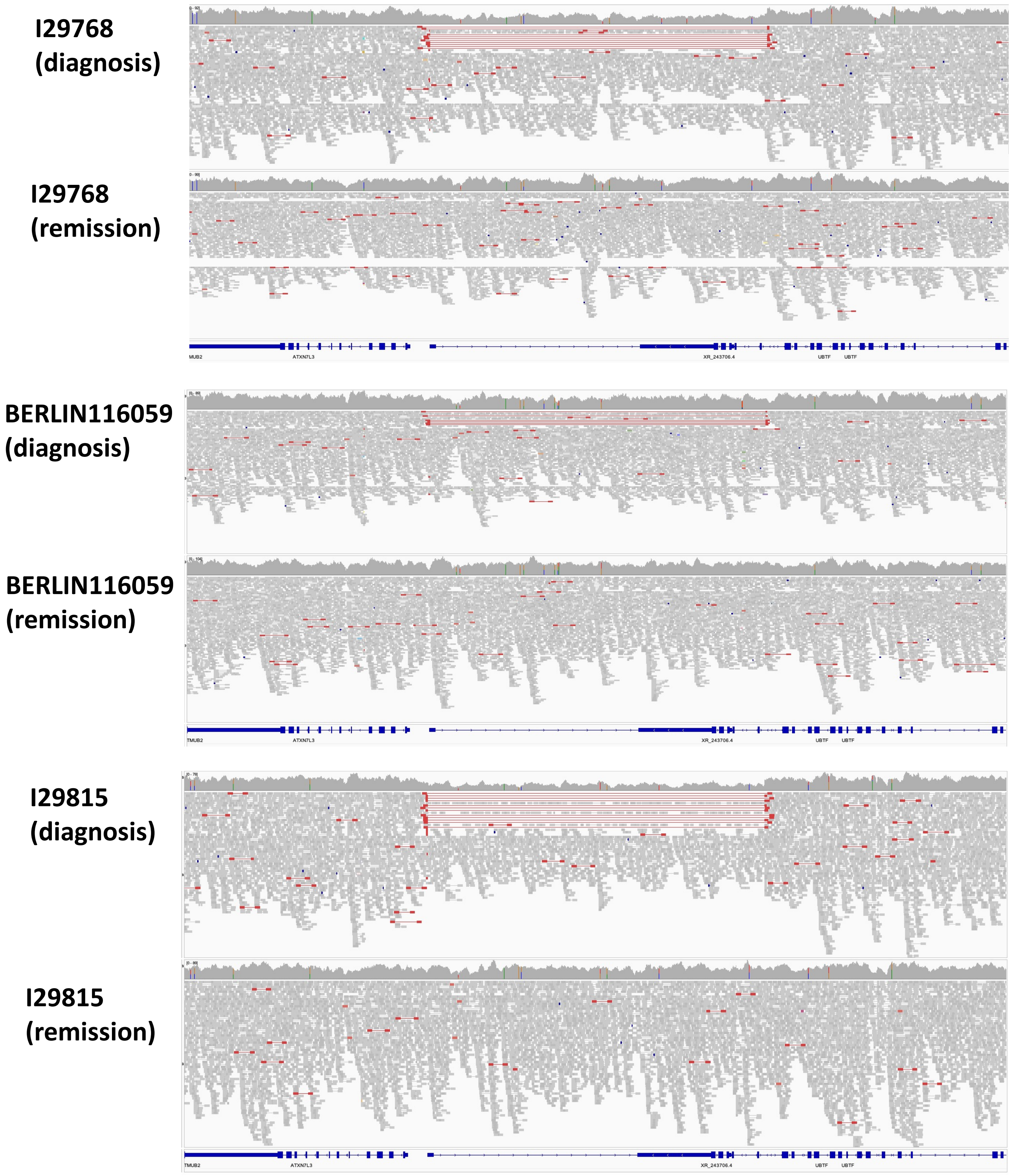

B

	deletion start	deletion end	pair count
**I29768**	chr17: 44198762	chr17: 44208837	10
**BERLIN116059**	chr17: 44198763	chr17: 44208844	6
**I29815**	chr17: 44198760	chr17: 44208837	12

Supplementary Figure S2: (A) WGS whole genome sequencing read alignment from n=3 UBTF-ATXN7L3 samples with reads depicted as pairs and red highlighting insert lengths above the 99.5 percentile of all reads (IGV Browser). Corresponding first diagnosis and remission samples are shwon. **(B**) Genomic coordinates (hg38) for the deletion breakpoints as called by MANTA.

Supplementary Figure S3

A

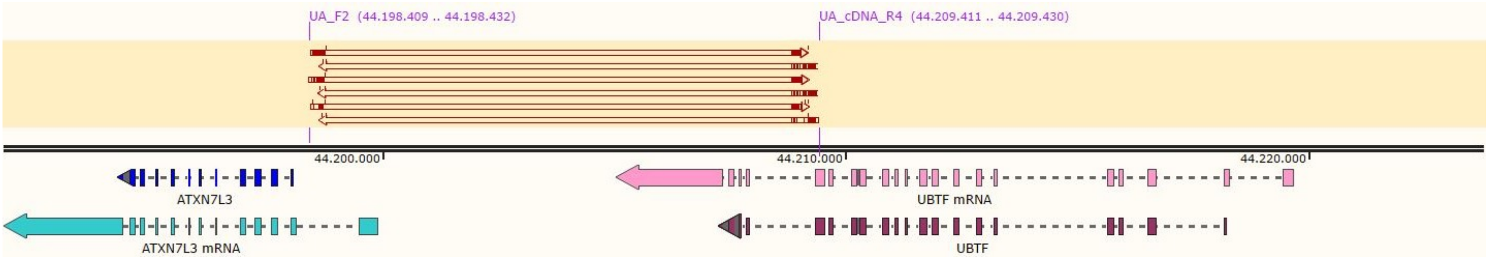

B

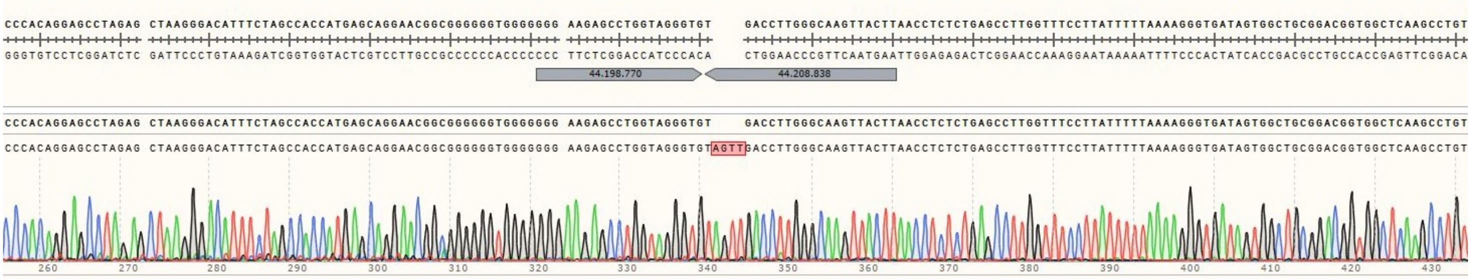

C

Primer	Sequence
UA_F2	CCTCTTTGATACCCATGGTGCAAT
UA_cDNA_R4	GAGCCAGAAGGAGCACTACA
Product length	949 bp

Supplementary Figure S3: (A) Alingment of representative sanger sequences from a deletion breakpoint-specific PCR to the same reference as uses in main figure 1. **(B)** Deletion break-point specific sanger sequence in one representative case. **(C)** Sequencing primers used to detect the deletion.

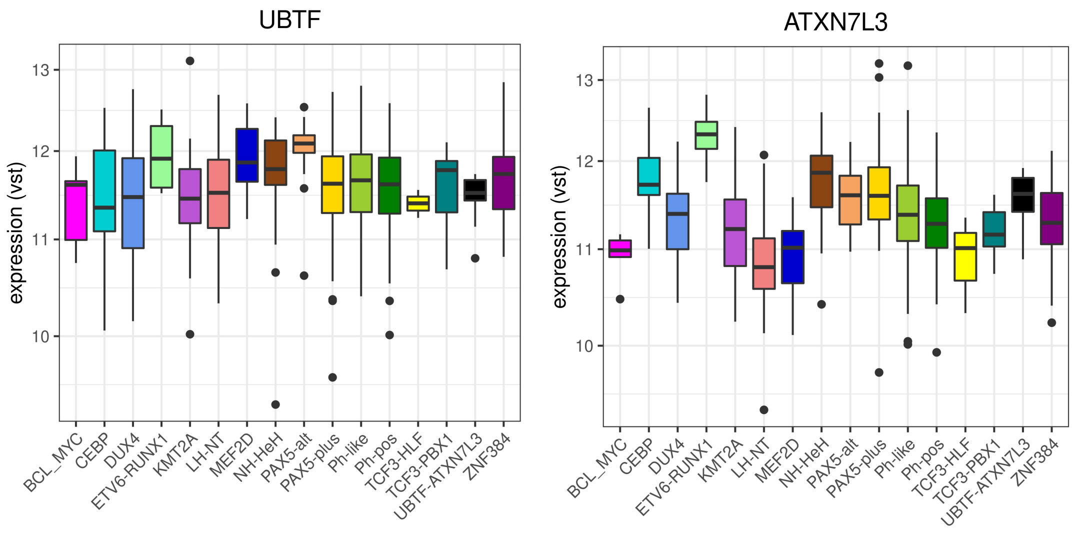

Supplementary Figure S4: Gene expression of *UBTF* and *ATXN7L* analyzed by RNA-Seq across all molecular subtypes of the described cohort.

Supplementary Figure S5

A

NAME	PUBLICATION	SIZE	ES	NES	NOM p-val	FDR q-val	
FABER_HSC-CDX_UP	*Faber K et al. J Clin Invest. 2013*	83	0.40307984	1.5061758	*0.012738854*	*0.06907962*	
VU_HSC UP AFTER CDX2_RESTRICTED	*Vu T el al. Nat Commun. 2020*	138	0.3223235	1.3047998	*0.026666667*	*0.13226715*	*
FABER_HSC-CDX_DN	*Faber K et al. J Clin Invest. 2013*	15	0.49183303	1.2725582	*0.18644068*	*0.11036589*	
CDX2_Q5_TRANSCRIPTION-FACTOR-BINDING-SITES	*Xie X, v7.4 TRANSFAC*	252	0.28819415	1.2559632	*0.033653848*	*0.09069534*	**
VU_CTRL-VS-B/T-ALL_UP	*Vu T el al. Nat Commun. 2020*	70	-0.27385712	-0.8899339	*0.65710187*	*0.85989803*	
LIU_CDX2_TARGETS_UP	*Liu T et al. Carcinogenesis. 2007*	35	-0.30903932	-0.8883947	*0.6210191*	*0.7915023*	
VU_CTRL-VS-AML_UP	*Vu T el al. Nat Commun. 2020*	1301	-0.2565115	-1.0669212	*0.21181263*	*0.3375884*	
GALLAND_CDX2_IN_HSC_UP	*Galland A et al. Mol Oncol. 2021*	252	-0.29893512	-1.1315628	*0.20387097*	*0.20987014*	
VU_CTRL-VS-PRE-LEUKEMIA_DOWN	*Vu T el al. Nat Commun. 2020*	1175	-0.284442	-1.1730226	*0.030927835*	*0.16104126*	
VU_CTRL-VS-B/T-ALL_DOWN	*Vu T el al. Nat Commun. 2020*	148	-0.34202182	-1.2298622	*0.09973046*	*0.10769993*	
VU_HSC DOWN AFTER CDX2_RESTRICETD	*Vu T el al. Nat Commun. 2020*	294	-0.32544467	-1.2498716	*0.043583535*	*0.104867905*	***
VU_HSC DOWN AFTER CDX2_LARGE	*Vu T el al. Nat Commun. 2020*	761	-0.30752006	-1.2520446	*0.014054054*	*0.124040164*	
VU_CTRL-VS-AML_DOWN	*Vu T el al. Nat Commun. 2020*	1706	-0.30926764	-1.2889386	*0.0*	*0.11436714*	
GALLAND_CDX2_IN_HSC_DN	*Galland A et al. Mol Oncol. 2021*	769	-0.3276253	-1.3411496	*0.0*	*0.10049875*	
VU_CTRL-VS-PRE-LEUKEMIA_UP	*Vu T el al. Nat Commun. 2020*	1522	-0.33282676	-1.3841629	*0.0*	*0.10596232*	
VU_HSC UP AFTER CDX2_LARGE	*Vu T el al. Nat Commun. 2020*	380	-0.38309363	-1.5052205	*0.0*	*0.08409579*	

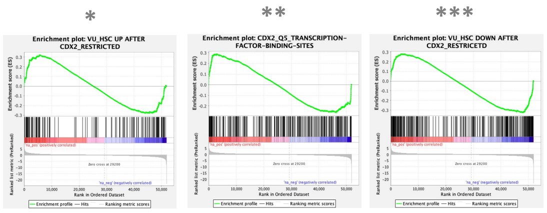

B

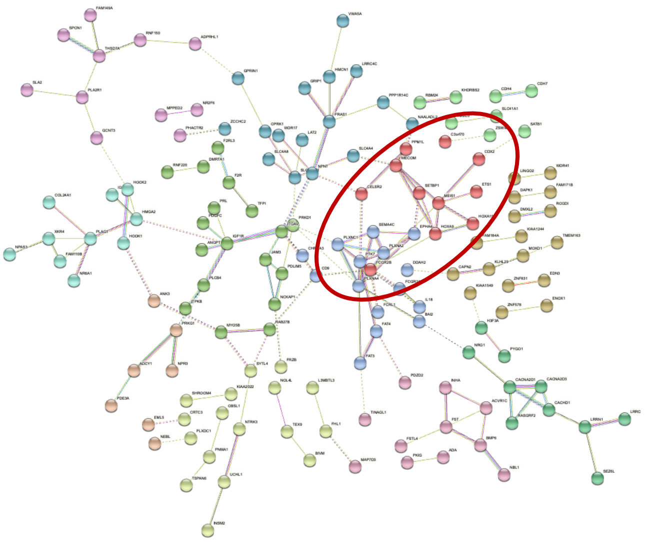

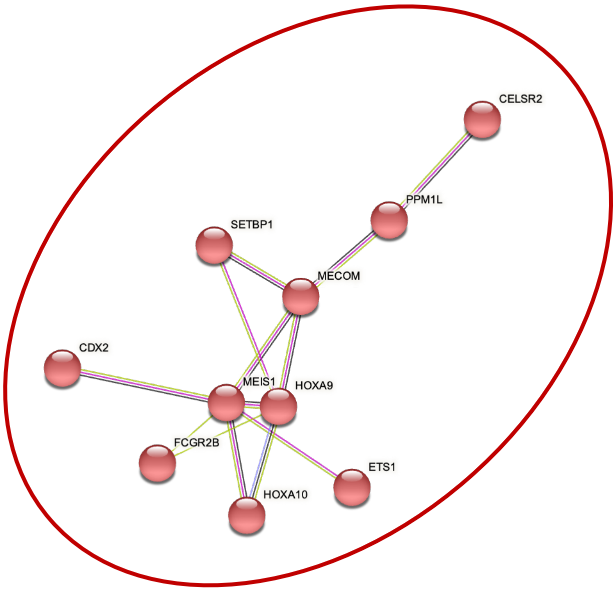

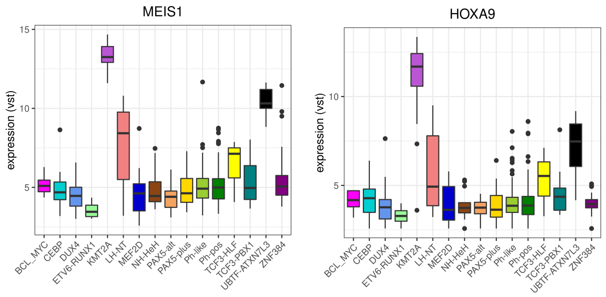

C

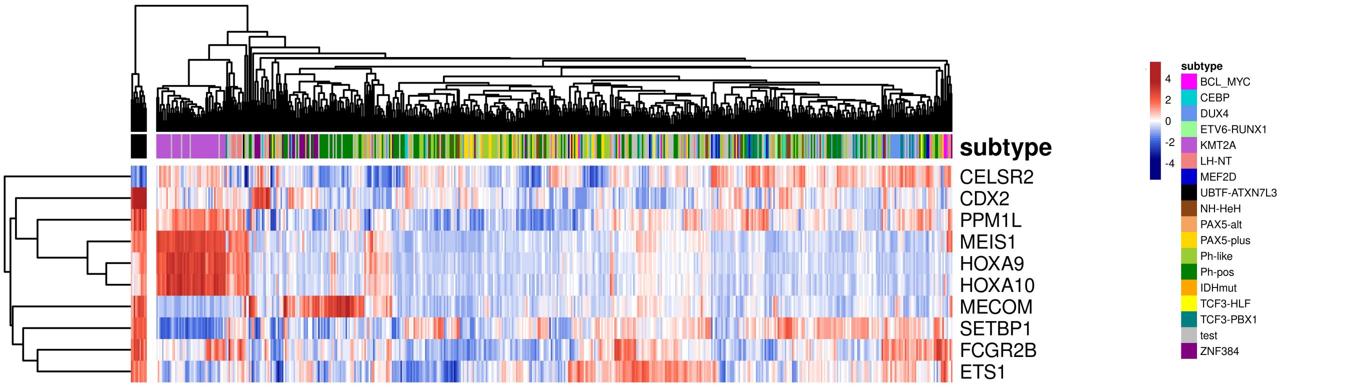

Supplementary Figure S5: (A) GESA preranked analysis comparing gene expression of UBTF-ATXN7L3 samples vs remaining ALL cohort to published models with conditional expression of Cdx2 in hematopoietics cells (Faber K et al. J Clin Invest. 2013, Vu T et al. Nat Commun. 2020, Galland A et al. Mol Oncol. 2021) or stable expression of CDX2 in esophageal epithelial cells (Liu T et al. Carcinogenesis. 2007) or CDX2 transcription factor targets from in-silico predictions (Xie x, v7.4 TRANSFAC). Representative GESA plots for selected analyses are shown. **(B)** UBTF-ATXN7L3 specific gene expression was established by multi-ANOVA comparison (Supplementary Table S2). STRING analysis (Szklarczyk D et al. Nucleic Acids Res. 2019) was used to identify fuctional modules employing k-means clustering. Genes with connections to at least one other network node are shown. Cluster 1 includes CDX2 in a context with MEIS1 and HOXA9, which both are upregulated in UBTF-ATXN7L3 ALL. **(C)** Heaptmap depicts expression of ‚cluster 1‘ genes across the entire cohort.

Supplementary Figure S6

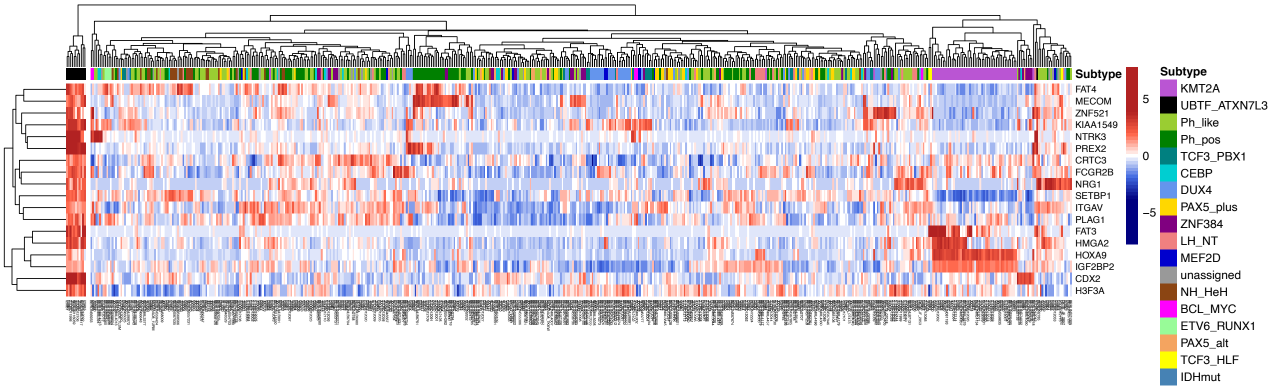

Supplementary Figure S6: Heatmap depicts unsupervised clustering of Cancer gene census genes upregulated in UBTF-ATXN7L patients compared to the remaining cohort.

Supplementary Figure S7

A

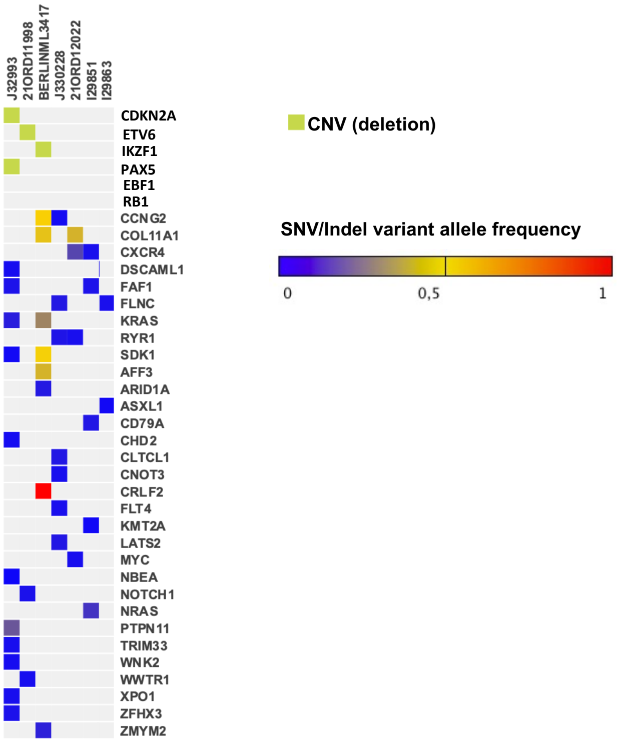

B

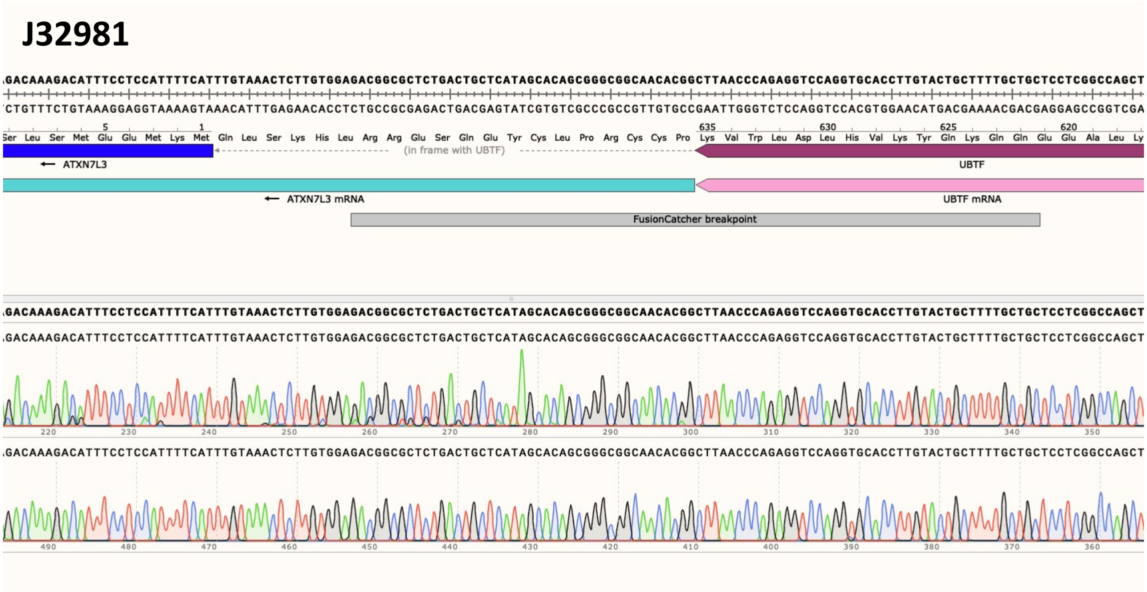

Supplementary Figure S7: (A) Heatmap depicting single nucleotide variants and insertions / deletions which either occurred recurrently in at least two samples or affected Cosmic cancer gene census genes. SAREK pipeline outputs were filtered to include only variants in protein coding regions with a MAX variant allele frequency <1E-04 in gnomAD and CADD-score >15. CNVs were analysed by SNP-arrays and / or CNVKit analysis of WES data in selected gens of interest. **(B)** Sanger sequencing results of break-point specific PCR for UBTF-ATXN7L3 in one patient of this subtype, where the gene fusion was not called from RNA-Seq data due to low blast percentage in the sample (20%).
